# Maastricht experience with the second generation endoscopic laser balloon ablation system for the atrial fibrillation treatment

**DOI:** 10.1007/s12471-015-0703-8

**Published:** 2015-06-02

**Authors:** N. Kumar, M.M. Abbas, R.M.A. Ter Bekke, C.M.M.J.F. de Jong, R. Choudhury, O. Bisht, S. Philippens, C. Timmermans

**Affiliations:** 1 Department of Cardiology, Maastricht University Medical Centre, and Cardiovascular Research Institute Maastricht (CARIM), P. Debyelaan 25, PO Box 5800, 6229 HX/6202AZ Maastricht, The Netherlands; 2 Department of Cardiology, Al Qassimi Hospital, Sharjah, U.A.E; 3 Department of Cardiology, AZ Sint Jan Brugge-Oostende AV, Brugge, Belgium; 4 Department of Cardiology, Heinrich-Braun-Klinikum Zwickau, Zwickau, Germany

Gal et al. [1] reported the results of the 50 patients treated for atrial fibrillation (AF) using the endoscopic laser balloon ablation system (EAS) in the Netherlands, who were recruited for the study between December 2011 and December 2013. The second-generation EAS uses a compliant balloon to improve balloon/tissue contact, which improves the energy delivery to create consistent lesions and, ultimately, to achieve durable pulmonary vein (PV) isolation. The study’s mean procedure and fluoroscopy time were 171 and 36 min, respectively. Temporary phrenic nerve palsy was observed during a single procedure. After a median follow-up of 17.3 (12.9–19.5) months, 58 % of the patients remained free of AF, after a single EAS PV isolation without the use of antiarrhythmic drugs.

Between November 2012 and July 2013, 24 patients underwent PV isolation using the second-generation EAS at the Maastricht University Medical Center. Oesophageal temperature monitoring was adopted for all patients to avoid undesired excessive oesophageal thermal injury. Acute PV isolation was achieved in all 80 PVs of 20 patients (100 %). The mean procedure and fluoroscopy time were comparable with that of Gal et al., 180 ± 44 and 25 ± 10 min, respectively. In all of these patients except four, a bolus of 15–21 mg of adenosine was injected followed by a rapid saline flush, 30 min after the last energy application [2]. Restoration of electrical conduction by adenosine-mediated hyperpolarisation occurred in 11 PVs (13.7 %) in seven patients (35 %), namely the left superior PV in four, the left inferior PV in two, the right superior PV in four, and the right inferior PV in one. Additional EAS-guided ablation was performed in these veins achieving complete isolation. We did not observe any oesophageal temperature rise above 39 °C for any of the patients. The complication rate was relatively low with one case of spontaneously resolving phrenic nerve palsy, one patient with transient ST-segment elevation conservatively managed by sublingual nitroglycerin, and one groin haematoma.

After a 3-month blanking period, patients received a 24–48 h Holter monitor every 3 months for 1 year to assess AF recurrence. Previously, we have reported that of these patients, 85 % were free of AF without antiarrhythmic drugs, after a median follow-up of 267 ± 76.9 days [3]. Currently, after a follow-up of 18.9 ± 2.3 months, the success rate is 81 % [4].

The higher medium-term AF-free survival after EAS-PV isolation at Maastricht may be partly explained by a potentially shorter learning curve due to prior experience with a similar balloon-based ablation system, i.e. cryoballoon. In addition, the elimination of adenosine-mediated PV reconnections may have contributed to the clinical benefit, although the incremental value remains to be resolved. In addition, elimination adenosine-mediated PV reconnections may have increased the overall success rate [5, 6].

## Funding

None.

## Conflict of interest

None declared.
